# Gut Protective Effect from D-Methionine or Butyric Acid against DSS and Carrageenan-Induced Ulcerative Colitis

**DOI:** 10.3390/molecules28114392

**Published:** 2023-05-28

**Authors:** Yuka Ikeda, Satoru Matsuda

**Affiliations:** Department of Food Science and Nutrition, Nara Women’s University, Kita-Uoya Nishimachi, Nara 630-8506, Japan; tyvufkxaq1226-218@outlook.jp

**Keywords:** inflammatory bowel disease, ulcerative colitis, D-methionine, D-amino acids, butyric acid, short chain fatty acids, postbiotics

## Abstract

Microbiome dysbiosis resulting in altered metabolite profiles may be associated with certain diseases, including inflammatory bowel diseases (IBD), which are characterized by active intestinal inflammation. Several studies have indicated the beneficial anti-inflammatory effect of metabolites from gut microbiota, such as short-chain fatty acids (SCFAs) and/or D-amino acids in IBD therapy, through orally administered dietary supplements. In the present study, the potential gut protective effects of d-methionine (D-Met) and/or butyric acid (BA) have been investigated in an IBD mouse model. We have also built an IBD mouse model, which was cost-effectively induced with low molecular weight DSS and kappa-carrageenan. Our findings revealed that D-Met and/or BA supplementation resulted in the attenuation of the disease condition as well as the suppression of several inflammation-related gene expressions in the IBD mouse model. The data shown here may suggest a promising therapeutic potential for improving symptoms of gut inflammation with an impact on IBD therapy. However, molecular metabolisms need to be further explored.

## 1. Introduction

The inflammatory bowel diseases (IBDs) are a kind of chronic inflammatory disorder of the gut. Ulcerative colitis (UC) and Crohn’s disease are the major types of IBD. UC is a recurrent inflammatory disease of the colon and rectum, characterized by intestinal mucosal ulceration, and infiltration of lymphocytes and/or neutrophils into the mucous membrane [1]. The morbidity of IBD has been globally increasing [2]. Genetic and/or environmental factors may be involved in its onset. However, the accurate mechanisms through which the beginning of IBD happens remain poorly comprehended [3]. Antioxidative and immunosuppressive reagents are used in IBD treatment; however, these therapies have been associated with severe side effects and long-term toxicity [4]. For example, novel therapeutic agents, such as tacrolimus, infliximab, and adalimumab, have become available to treat UC [5]. However, many of the therapeutic agents are immune suppressants, which makes the risk of infection increase [6]. Therefore, it is important to explore safe and/or actual regimens, such as dietary interventions to complement IBD therapies. Many studies have suggested that IBD might be closely related to an alteration of the gut microbiota composition and/or destruction of the intestinal barrier [7]. However, the underlying mechanisms of this have not been well elucidated yet.

**Figure 1:**
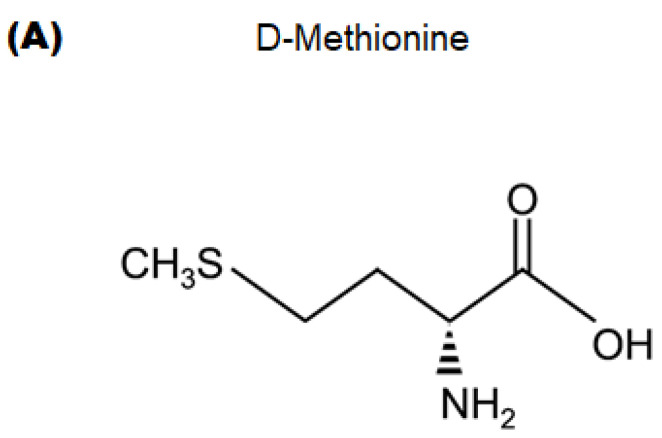

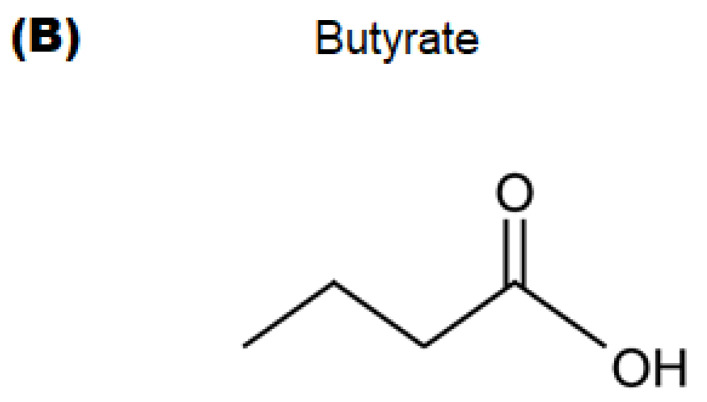
Structural formula of D-methionine (**A**) and butyric acid (**B**).

IBD is deliberated to result from an unsuitable immune response to the intestinal microflora [8]. The resulting inflammation induces tissue damage and the disruption of the epithelial barrier, leading to the perturbation of the intestinal microenvironment, which may cause a relationship between the mucosal surface of IBD and the commensal microbiota. The disequilibrium in the gut may also result in the disquiet of the composition of commensal microbiota, leading to dysbiosis [9]. In addition, inflammation may induce tissue damage and a disruption of the epithelial barrier leading to the perturbation of the intestinal microenvironment, which is related to the commensal microbiota [7]. Microbiota in IBD patients may be particularly unstable compared to those in healthy individuals [8]. Therefore, gut microbiota may be a promising therapeutic target for IBD [7,8]. In fact, it has been shown that certain probiotics could serve as a mediator of anti-inflammation in IBD to restore intestinal microbiota composition [10]. Similarly, the colitis symptoms have been alleviated by the probiotic bacteria administration, which might also mitigate the gut epithelial barrier damage [11]. Biological and/or biological mechanisms of probiotics with health-beneficial effects remain unknown. Certain probiotics may inhibit the growth of pathogenic microorganisms. Interestingly, recent studies have recognized that a range of metabolites produced by the fermentation of gut microbiota may be useful to host health, which may include SCFAs such as acetic acid, propionic acid, and/or butyric acid [12]. The concept of postbiotics has been proposed by the relationship with the valuable effects of probiotics, which may also convey a considerable health benefit to the host [13]. Postbiotics may represent an alternative option when referring to biological approaches for the malady host, as their application could be safer compared to functional products of living microorganisms [14]. However, it would become a complex mixture that may include the components of non-living microorganisms, peptides, proteins, enzymes, SCFAs, minerals, and/or vitamins. In addition, it has been shown that D-amino acids are also metabolites with a protective role for several organs, produced by the fermentation in the gut microbiota [15]. Initially, the aim of this study was to evaluate the protective effect of beneficial gut microbiota-derived postbiotics, such as D-amino acids and/or SCFAs. After several preliminary experiments, we hypothesized that supplementation of either D-methionine and/or butyric acid as postbiotics could improve the pathology of IBD. Dextran sulfate sodium (DSS) and/or carrageenan could induce severe intestinal mucosal inflammation and colitis, which may be considered a type of UC model in terms of morphological and pathophysiological features [16]. These animal models have been generally used to understand the molecular mechanisms of action or to assess the therapeutic effects of several compounds in the treatment of UC [17]. In our preliminary experiments, we used several D-amino acids and/or various short-chain fatty acids, including D-leucine, D-proline, D-tryptophan, acetic acid, propionic acid, etc., for the treatment of UC. Among them, the promising effects were repetitively found in D-methionine and/or in butyric acid. Therefore, in the present study, we evaluated the effects of both D-methionine and butyric acid, one of the SCFAs, on the development of DSS and carrageenan-induced experimental colitis in mice. The structural formulas of d-methionine and butyrate are shown in Figure 1. In conclusion, we found that both could improve the pathology of IBD in an animal model.

## 2. Results

### 2.1. Characteristics and Disease Activity Index of UC Model Mice

**Figure 2:**
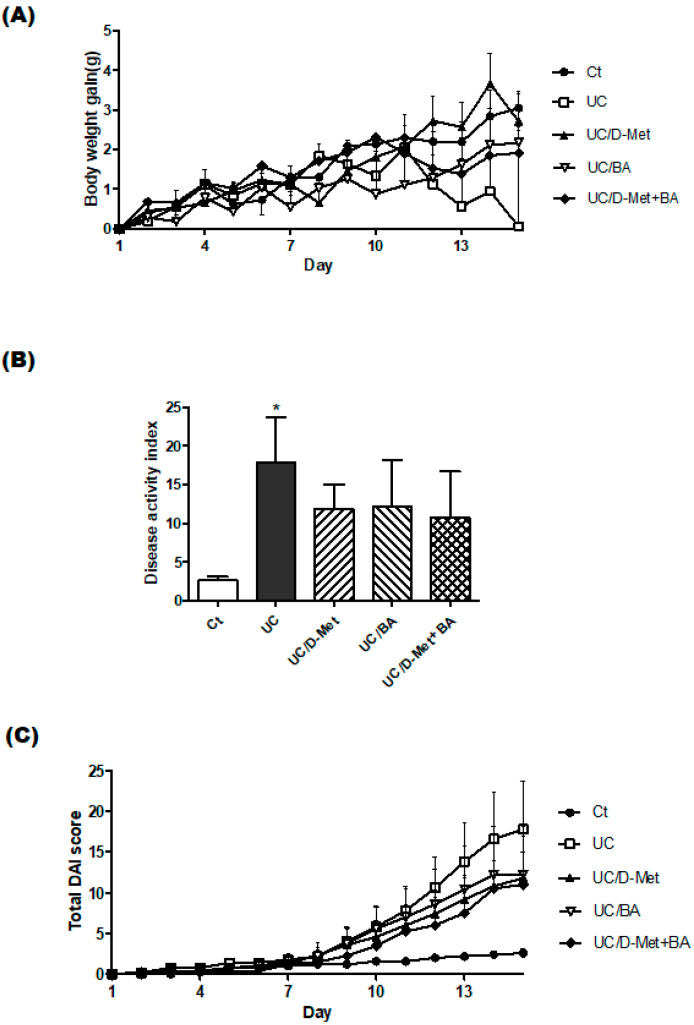
Effects of D-methionine (D-Met) and butyric acid (BA) intake on body weight loss and disease activity index. (**A**) Body weights were measured every day during the experiment. Ct group (black circle), UC group (white square), UC/D-Met group (black triangle), UC/BA group (white inverted triangle), and UC/D-Met + BA group (black diamond). Values are expressed as the mean ± SE, *n* = 5/group. The data were tested by two-way ANOVA. (**B**) Disease activity index is the score that sums the daily score of five grades of weight loss and stool consistency and four grades of occult blood. Ct group (white), UC group (black), UC/D-Met group (right upper diagonal), UC/BA group (left upper diagonal), and UC/D-Met + BA group (mesh pattern). Values are expressed as the mean ± SE, *n* = 5/group. The data were tested by t-test. (* *p* < 0.05, vs. Ct group). (**C**) Disease activity index scores were summed every day during the experiment. Ct group (black circle), UC group (white square), UC/D-Met group (black triangle), UC/BA group (white inverted triangle), and UC/D-Met + BA group (black diamond). Values are expressed as the mean ± SE, *n* = 5/group. The data were tested by two-way ANOVA.

Body weight may be a major clinical feature of IBD [18]. We recorded the changes in body weights to investigate the benefit of D-methionine or butyrate on weight gain, which is described in Figure 2. In the first 4–5 days, all mice grew almost equally, and their stools were normal. However, after day 7, the mice, except for the Ct group, gradually exhibited some stool abnormalities, such as bloody and soft stools. At the same time, the body weight gain rate declined. After day 12, the body weight of the UC group mice considerably decreased (Figure 2A). As shown in Figure 2A, the body weight gain of mice on day 14 was significantly lower in the UC group than in the Ct group. No mice died during the entire experiment. The body weights for the orally administered D-Met group were somewhat higher compared to the butyrate group. The levels of body weight in the UC group were significantly lower than in the D-Met or BA group that had the additional treatment, which means the UC mice without the D-Met and/or BA treatments could cause a significant loss in body weight. In addition, severe rectal bleeding and diarrhea were observed. The body weight loss and symptoms of stool were scored and shown in Figure 2B. UC group was the highest score of disease activity index (DAI) (*p* < 0.05 vs. Ct), and UC/D-Met, UC/BA, and UC/D-Met + BA groups were higher than in the Ct group, were lower than in the UC group (Figure 2B). The daily alterations in DAI score are also shown (Figure 2C).

**Figure 3:**
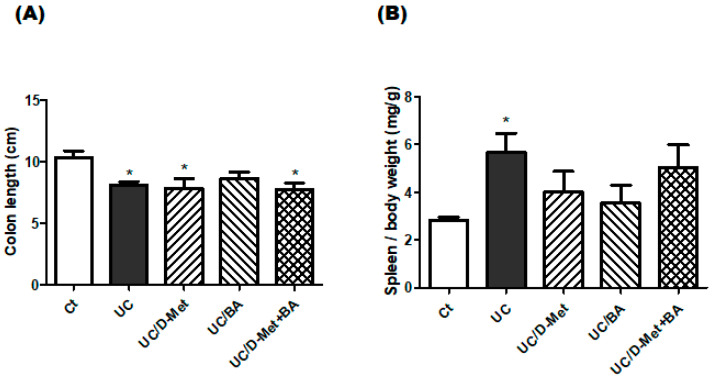
**Colon lengths and spleen weights in mice:** (**A**) Colon lengths were measured when mice were dissected. Ct group (white), UC group (black), UC/D-Met group (right upper diagonal), UC/BA group (left upper diagonal), and UC/D-Met + BA group (mesh pattern). Values are expressed as the mean ± SE, *n* = 5/group. The data were tested by one-way ANOVA. (* *p* < 0.05, vs. Ct group). (**B**) Relative spleen weights were measured when mice were dissected. Ct group (white), UC group (black), UC/D-Met group (right upper diagonal), UC/BA group (left upper diagonal), and UC/D-Met + BA group (mesh pattern). Values are expressed as the mean ± SE, *n* = 5/group. The data were tested by *t*-test. (* *p* < 0.05, vs. Ct group).

One of the characteristics of UC model mice is the shorter length of the colon [19]. Morphology analysis of the colon may show that the colon lengths of mice in the Ct group would be significantly longer than those in the DSS group mice. Consistently, the colon length of all UC-induced groups was shorter than in the Ct group. However, there was no difference between the UC and the UC + additional treatment with D-Met and/or BA groups in terms of colon length (Figure 3A). In addition, the spleen weight of the UC model mice may be increased [20]. Consistently, the spleen weights of the UC-induced group were higher than in the Ct group. The spleen weight in the UC group was significantly higher than that in the control group (*p* < 0.05), although it relatively decreased after supplementation with D-methionine or butyrate (Figure 3B). In particular, the spleen weights of the UC and UC/ D-Met + butyrate groups were higher than in the UC/D-Met and/or UC/BA groups (Figure 3B). There was almost no difference in the brain weights in the groups.

### 2.2. Histological Finding in the Colon

**Figure 4:**
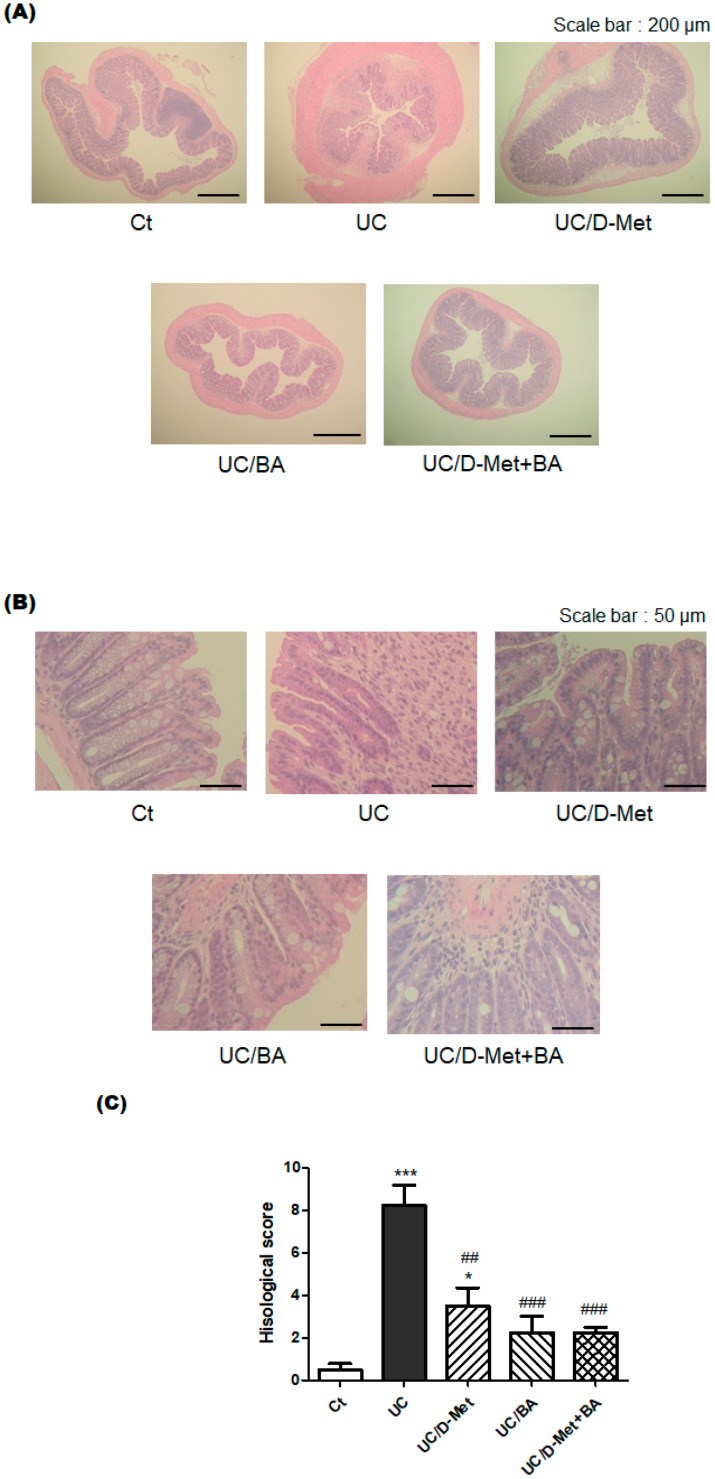
Histopathological analysis in the colon: (**A**) HE staining of mice colon tissues. The magnification was ×200. Each scale bar shows 200 µm. (**B**) HE staining of mice colon tissues. The magnification was ×800. Each scale bar shows 50 µm. (**C**) Histological scores are shown, which are calculated with the score of four grades in epithelium loss, crypt damage, depletion of goblet cells, and infiltration of inflammatory cells for Ct group (white), UC group (black), UC/D-Met group (right upper diagonal), UC/BA group (left upper diagonal), and UC/D-Met + BA group (mesh pattern). Values are expressed as the mean ± SE, *n* = 5/group. The data were tested by one-way ANOVA. (*** *p* < 0.005, * *p* < 0.05, vs. Ct group and ^###^ *p* < 0.005, ^##^ *p* < 0.01, vs. UC group).

Next, we investigated the effect of D-methionine or butyric acid on the histological damage in the colon using HE staining. UC may be characterized by histological findings, such as edema, infiltration of inflammatory cells into the mucosa and submucosa, destruction of epithelial cells, and mucosal thickening. The pathophysiology of UC is the disturbance of intestinal epithelial cells, lymphocyte infiltration, edema, narrowing of the intestinal canal, and thickened colonic muscular layer. Consistently, our microscopic analysis also found that colon epithelial cells in the UC mice were severely damaged (Figure 4). Figure 4B shows the increased magnification of the sample images illustrated in Figure 4A. Infiltration of inflammatory cells into the submucosa was also observed in the colon epithelial cells in the UC group. Furthermore, edematous findings were also observed especially in the submucosa of UC and/or UC/D-Met groups compared to the Ct group. Whereas the intestinal crypts of the Ct group were orderly arranged in a row and spaced closely, those of the UC group were messily arranged with wide spaces. In addition, the intestinal tract of the UC group was narrow, and the muscular layer of the UC group was seriously thickened, showing that intestinal epithelial cells in the UC group were severely damaged (Figure 4A,B). We used the combination of low molecular weight DSS and kappa carrageenan for making the UC group mouse model. Consequently, they could cost-effectively build a mice model of IBD, as shown here. It has been observed that D-Met and butyrate might have protective effects on the gut injury of these UC model mice (Figure 4C).

### 2.3. Analyses for Genes and Protein Expression in the Colon of UC Mice

**Figure 5:**
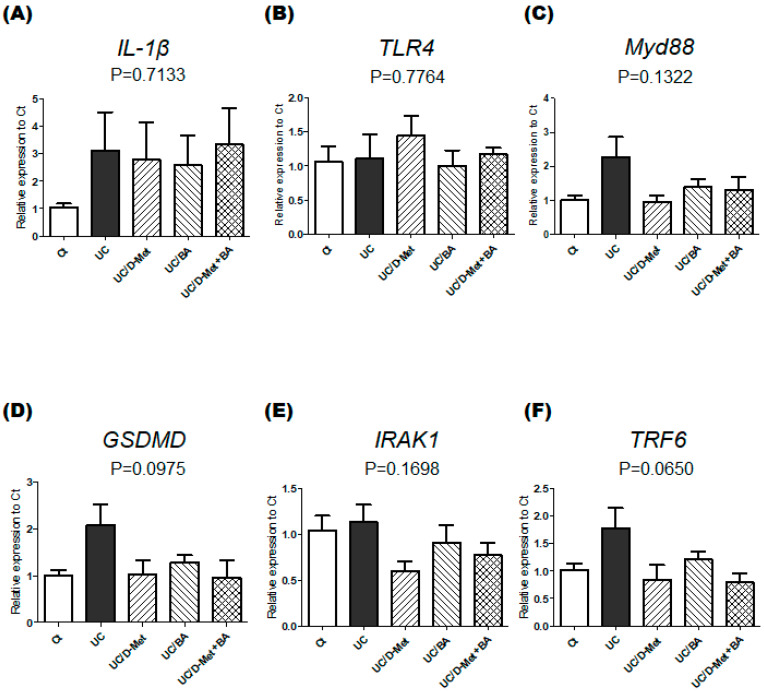

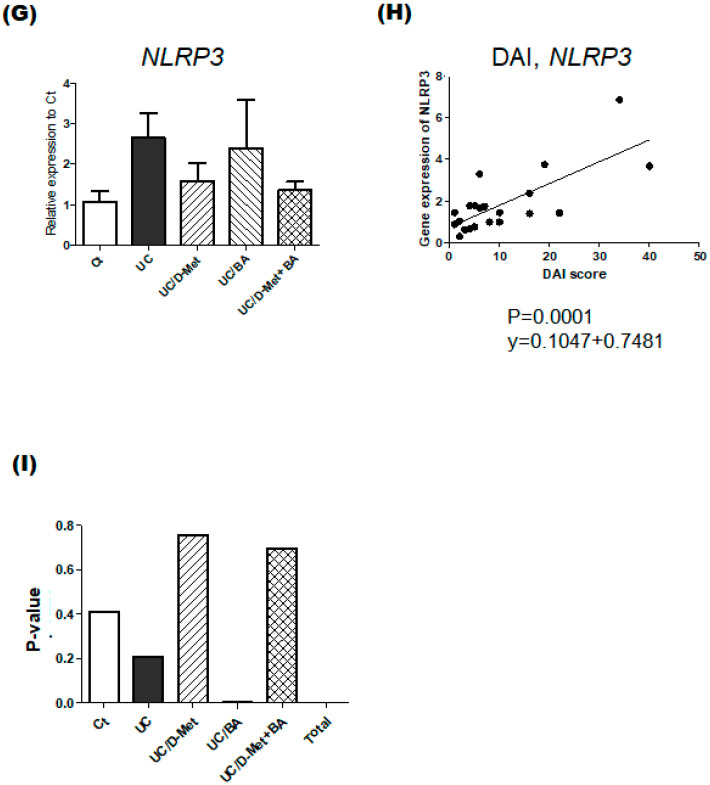
(**A**–**F**). The mRNA expression in the colon: (**A**) The mRNA expression of *IL-1β* was measured and normalized to β-actin by RT-PCR. Ct group (white), UC group (black), UC/D-Met group (right upper diagonal), UC/BA group (left upper diagonal), and UC/D-Met + BA group (mesh pattern). Values are expressed as the mean ± SE, *n* = 5/group. The data were tested by one-way ANOVA. IL-1β: interleukin 1β. (**B**) The mRNA expression of *TLR4* was measured and normalized to β-actin by RT-PCR. Ct group (white), UC group (black), UC/D-Met group (right upper diagonal), UC/BA group (left upper diagonal), and UC/D-Met + BA group (mesh pattern). Values are expressed as the mean ± SE, *n* = 5/group. The data were tested by one-way ANOVA. TLR4: toll-like receptor 4. (**C**) The mRNA expression of *MyD88* was measured and normalized to β-actin by RT-PCR. Ct group (white), UC group (black), UC/D-Met group (right upper diagonal), UC/BA group (left upper diagonal), and UC/D-Met + BA group (mesh pattern). Values are expressed as the mean ± SE, *n* = 5/group. The data were tested by one-way ANOVA. MyD88: myeloid differentiation factor 88. (**D**) The mRNA expression of *GSDMD* was measured and normalized to β-actin by RT-PCR. Ct group (white), UC group (black), UC/D-Met group (right upper diagonal), UC/BA group (left upper diagonal), and UC/D-Met + BA group (mesh pattern). Values are expressed as the mean ± SE, *n* = 5/group. The data were tested by one-way ANOVA. GSDMD: gasdermin D. (**E**) The mRNA expression of *IRAK1* was measured and normalized to β-actin by RT-PCR. Ct group (white), UC group (black), UC/D-Met group (right upper diagonal), UC/BA group (left upper diagonal), and UC/D-Met + BA group (mesh pattern). Values are expressed as the mean ± SE, *n* = 5/group. The data were tested by one-way ANOVA. IRAK1: interleukin 1 receptor-associated kinase 1. (**F**) The mRNA expression of *TRF6* was measured and normalized to β-actin by RT-PCR. Ct group (white), UC group (black), UC/D-Met group (right upper diagonal), UC/BA group (left upper diagonal), and UC/D-Met + BA group (mesh pattern). Values are expressed as the mean ± SE, *n* = 5/group. The data were tested by one-way ANOVA. TRAF6: TNF receptor-associated factor 6. (**G**–**I**). **The correlation of the *NLRP3* gene expression to the DAI score**: (**G**) The mRNA expression of *NLRP3* was measured and normalized to β-actin by RT-PCR. Ct group (white), UC group (black), UC/D-Met group (right upper diagonal), UC/BA group (left upper diagonal), and UC/D-Met + BA group (mesh pattern). Values are expressed as the mean ± SE, *n* = 5/group. The data were tested by one-way ANOVA. NLRP3: NLR family pyrin domain containing 3. (H) Positive correlation between the *NLRP3* mRNA expression normalized to β-actin and the DAI score. The *NLRP3* mRNA expression is strongly related to DAI score. y = 0.1047 x + 0.7481, *p* = 0.001. *n* = 21. The data were tested by Pearson’s correlation analysis. (I) Each group’s *p*-value of positive correlation between the *NLRP3* mRNA expression normalized to β-actin and the DAI score. Ct group (white), UC group (black), UC/D-Met group (right upper diagonal), UC/BA group (left upper diagonal), and UC/D-Met + BA group (mesh pattern). *n* = 5/group.

The proinflammatory cytokine interleukin 1β (IL-1β) has been postulated as a pathogenic factor in IBD [21]. The gene expression of IL-1β in the UC group was significantly higher than in the Ct group, which might be unaffected by the treatment of D-Met and/or butyrate (Figure 5A). Meanwhile, there was almost no difference in the gene expression of toll-like receptor 4 (TLR4) among all groups (Figure 5B). TLR4 is an important member of the TLRs family, which are the first mechanisms that recognize inflammation transducing the inflammation signal via the myeloid differentiation factor 88 (Myd88)-dependent pathway [22]. Consequently, a number of inflammasomes might activate the substrate proteins, including gasdermin D (GSDMD), in the inflammatory response [23]. In the meantime, the IL-1 receptor-associated kinase (IRAK1) and tumor necrosis factor (TNF) receptor-associated factor 6 (TRAF6)-mediated inflammatory signaling pathway might also be involved in the pathogenesis of UC [24]. In the gene expressions of Myd88, GSDMD, IRAK1, and TRAF6 in the present experiment, the expressions of the UC group were relatively higher than those of the Ct group. However, the elevation of the gene expressions appeared to be inverted to a certain level with the treatment of D-Met and/or butyrate (Figure 5C–F).

Nod-like receptor (NLR) family pyrin domain containing 3 (NLRP3) has been identified as a binding partner of the autophagy inhibitor mTOR [25]. NLRP3 could inhibit autophagy, impairing the autophagy-mediated elimination of proinflammatory mediators, which could consequently exacerbate inflammation. In fact, an anti-inflammatory effect on DSS-induced colitis in mice has been reported by inhibiting the NLRP3 inflammasome [26]. The pattern of NLRP3 gene expression was similar to IRAK1 or TRF6 gene expressions, showing that D-methionine or butyric acid could inhibit the expression of NLRP3 (Figure 5G). Interestingly, the NLRP3 gene expression was positively correlated to the DAI score (R = 0.7458, *p* = 0.0001) (Figure 5H). Figure 5I shows *p*-values for the correlation between the *NLRP3* gene expression and the DAI score within different subgroups. Amazingly, the *p*-value of the UC/BA group was the lowest and only significant among the subgroups.

**Figure 6:**
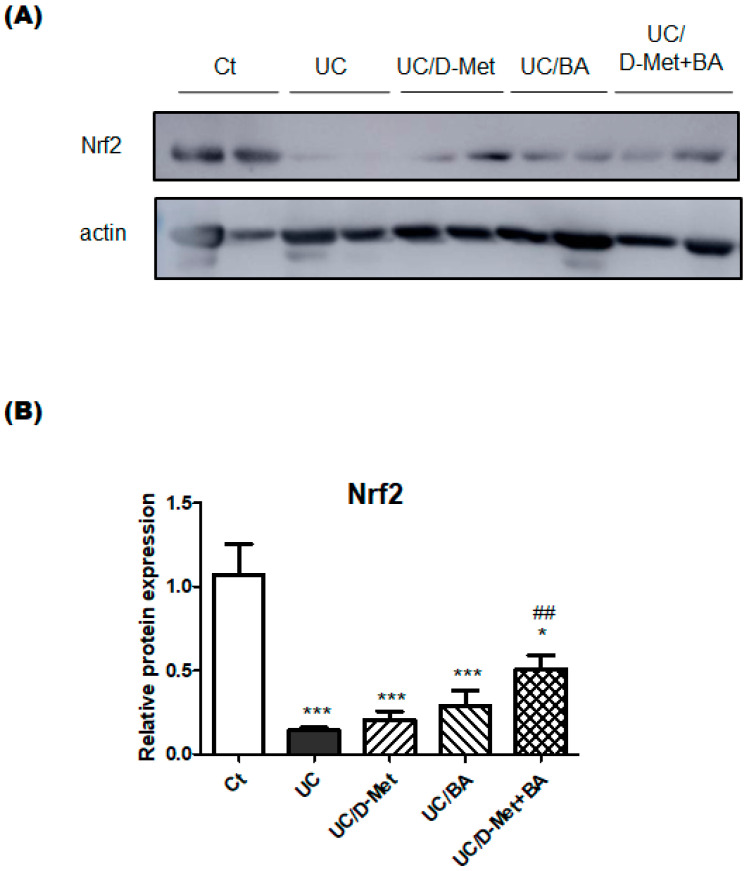
The expression of antioxidant enzymes in the colon: (**A**) The image of Nrf2 expression. (**B**) The protein expression of Nrf2 was measured and normalized to β-actin by Western blot. Ct group (white), UC group (black), UC/D-Met group (right upper diagonal), UC/BA group (left upper diagonal), and UC/D-Met + BA group (mesh pattern). Values are expressed as the mean ± SE, *n* = 5/group. The data were tested by one-way ANOVA. Nrf2: nuclear factor-erythroid 2-related factor 2. (* *p* < 0.05, *** *p* < 0.005, vs. Ct group, ^##^ *p* < 0.01, vs. UC group).

IBD may be closely linked to the imbalance of oxidative stress. Therefore, excessive amounts of reactive oxygen species (ROS) might also be produced by neutrophils and/or macrophages in the gut of inflammation of mice models. At the molecular level, UC is connected with the nuclear factor-erythroid 2-related factor-2 (Nrf2), which is a key factor in the oxidative stress response signaling pathway [27]. The activation of the Nrf2 pathway plays an important role in cellular redox status and oxidative stress [28]. In this experiment, Western blotting showed that the protein expression of NRF2 was also obviously downregulated in the UC group compared to the control group, which was slightly reversed by the treatment of D-Met and butyric acid, suggesting that D-Met and butyric acid may have a mild anti-inflammatory effect via NRF2 signaling (Figure 6).

## 3. Discussion

Although multiple factors, such as environmental changes, gene variations, and gut microbiota, were thought to be associated with UC, its pathogenesis has not been fully elucidated [29]. Disruption of the mucosal immune response produces excess amounts of inflammatory cytokines and matrix metalloproteinase, and also increases oxidative stress, resulting in severe damage to the intestine [30]. The present study aimed to explore the colon-protective effects of D-methionine and/or butyrate in a mouse model of UC. Injection of DSS or carrageenan could cause ROS accumulation and inflammatory response in gut epithelial cells [31,32]. In addition, the disruption of the gut epithelium might be associated with the elevated lipopolysaccharide (LPS) level [33]. The previous results have shown that chemotherapy-associated gastrointestinal toxicity is related to the increased relative abundance of LPS-producing bacteria [34].

In the present study, we observed the protective effects of D-Met and/or butyrate on gut injury in an UC mouse model. For example, D-Met and/or butyrate considerably relieved the severity in the UC mice (Figure 2). However, there was no difference in the colon length between the UC group and UC plus D-Met and/or butyrate groups. However, it would probably take more time to recover the colon damage until the full recovery of the length. In an innate immune system, TLRs are key factors, which could transmit the signal of inflammatory reaction through MyD88 dependent pathway. Consequently, the signal may mediate the expression of inflammatory factors, then, facilitate the occurrence of an inflammatory injury [35]. As a transcription factor, *Nrf2* could bind antioxidant response elements and might regulate the corresponding downstream genes, such as antioxidant enzyme genes [36]. The signaling pathway of Nrf2 seems to be a cellular mechanism to counteract ROS and/or prevent oxidative stresses, which could suppress the local production of proinflammatory cytokines in the surrounding intestinal cells. In fact, *Nrf2* signaling has been found to have antioxidative and anti-inflammatory effects in many organs. It has been reported that the protein expression of Nrf2 in DSS-exposed mice was significantly reduced due to chronic colitis [37], which might be in line with the result of the present experiment (Figure 6). The treatment with D-Met and/or butyrate could increase the expression of Nrf2, as shown in Figure 6. Similarly, several studies have shown that polyphenols have the potential to activate Nrf2 and in turn, enhance the expression of antioxidative genes in the gut [38]. In addition, the activation of the *Nrf2* signaling pathway has a positive effect on a variety of inflammatory diseases [39,40]. These may explain how butyrate could decrease oxidative stress and/or injury in the gut epithelial cells, which is consistent with a previous study [41]. Therefore, the possible beneficial effects of *L. reuteri* or butyric acid may be associated with anti-inflammatory and/or antioxidative effects [42]. Similarly, *Clostridium butyricum (C. butyricum)* could also ameliorate inflammation in the colon by decreasing the levels of proinflammatory cytokines [43] and increasing the anti-inflammatory metabolites of lipids [44]. *C. butyricum* can enhance the butyric acid production, which might protect the intestinal epithelia decreasing the oxidative damage of epithelium [44]. *C. butyricum* can produce several SCFAs, including butyric acid, which may have beneficial effects for the protection of several organs. In particular, butyrate could employ the modification of the gut microbial community [33,45], which could also regulate the mucosal immune response [45,46,47]. In addition, butyrate can not only inhibit pathogenic bacteria while stimulating the growth of beneficial bacteria, such as SCFAs-producing bacteria [48], yet could also decrease the levels of microbiota-dependent endotoxins such as LPS [49]. Moreover, butyrate-producing bacteria may alleviate intestinal permeability for reducing systemic toxins [50]. In these ways, probiotics and/or postbiotics relevant to the action of butyrate seem to be considered effective protectants of host organs, which could improve the pathology of IBD.

It has been shown that mice treated with D-amino acids prior to the induction of inflammatory colitis have exhibited a considerable reduction in inflammation that is not seen in mice fed with the corresponding L-amino acids [51]. Therefore, D-amino acids may have active properties as a prevention and/or a treatment for inflammation [51]. Moreover, several studies have shown the suggestion of D-amino acids in clinical practice [52]. For example, D-methionine can defend against intestinal damage via its antioxidative and/or anti-inflammatory properties, which could also adjust the imbalance of gut microbiota by increasing the growth of advantageous bacteria [53]. The protective effects of D-serine have been shown to inhibit oxidative damage [54]. In addition, D-cysteine administration might be useful for the treatment of several inflammatory diseases [55]. Furthermore, the protective effect of D-cysteine but not of L-cysteine has been shown via the effects of decreasing cellular damage, edema, and/or apoptosis of epithelium [56]. Treatment with D-aspartate may also provide helpful effects on the nervous system [57]. These data suggest that D-amino acids could have beneficial and/or protective effects on various organs with inflammation, which might be favorable to the prevention and/or treatment of several inflammatory diseases. In general, the D-amino acids are essential for an element of peptidoglycan in the cell wall of bacteria. Accordingly, gut microbiota may have a large genetic capacity to produce various D-amino acids for supporting bacterial growth [58]. In fact, many bacterial species encode specific racemases that can convert L-amino acids to D-amino acids, which are frequently present in peptidoglycan-containing bacteria [59]. Therefore, higher levels of D-amino acids may be associated with the mass of gut microbiota [60]. Consequently, the metabolism of D-amino acids in the host may be altered by the modification of gut microbiota [61]. Interestingly, the reduction in several D-amino acids may promote senescence through the increase in reactive oxygen species (ROS) production [62,63]. Our results are basically consistent with those published results. However, the reason why D-Met or BA could narrowly increase the expressions of Nrf2 remains unknown. We also cannot rule out the potential of multiple mechanisms contributing to the protective roles of D-Met and/or BA in the present experimental setting.

The possibility of using NLRP3 inhibitors has been shown to be potential therapeutic molecules for IBD treatment. For example, agonist-mediated activation of G-protein-coupled receptors (GPCRs) may inhibit the NLRP3 inflammasome activation, which could attenuate the DSS-induced colitis in mice [64]. In addition, it has been reported that the administration of an herbal extract to mice with DSS-induced colitis enhances mitophagy, leading to NLRP3 inflammasome inhibition, and subsequently the amelioration of colitis [65]. Consistent with this, our results also demonstrated that the NLRP3 gene expression in the present study was positively correlated to the DAI score (Figure 5G,H). However, therapies involving the modulation of autophagy and NLRP3 inflammasome to alleviate IBD should be more explored. In particular, it would be interesting to explore the molecular mechanisms by which butyric acid might link the *NLRP3* gene expression to the DAI score (Figure 5I).

Some limitations to this study should be noted. In particular, the small sample size of this study may lead to non-significant results. Therefore, the results of the analyses in a small sample size study should be interpreted with caution. For example, we cannot rule out the possibility that the results of this study overemphasized the correctness due to the small number of mice used in each subgroup. In addition, as the results of this study have been obtained by using a mouse model, the generalizability of our findings to humans should be carefully assessed. Future studies using a larger number of animals to address the above concerns would be informative.

## 4. Materials and Methods

### 4.1. Mice and Water

**Figure 7:**
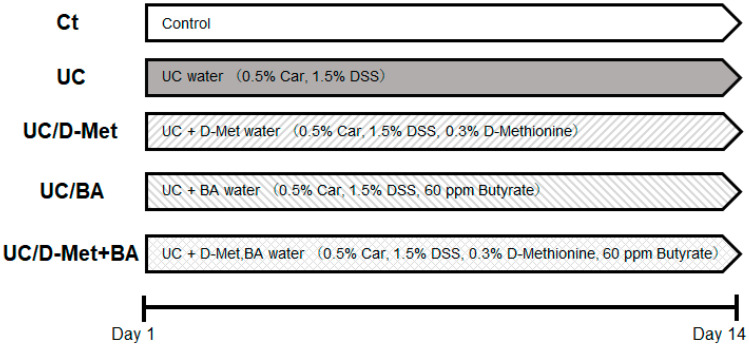
Study design: ICR mice (6 weeks old) were divided into five groups of Ct (control), UC (ulcerative colitis), UC/D-Met (ulcerative colitis/D-methionine), UC/BA (ulcerative colitis/butyric acid), U/D-Met + BA (ulcerative colitis/D-methionine + butyric acid) and sacrificed on day 14. DSS: dextran sodium sulfate; Car: κ-carrageenan.

Six-week-old male ICR mice were purchased from Charles River Laboratories Japan, Inc. (Kanagawa, Japan). In week one, we acclimatized the timings, and after that, the mice were randomly divided into five groups: Ct (control), UC (ulcerative colitis), UC/D-Met (ulcerative colitis/D-methionine), UC/BA (ulcerative colitis/butyrate), U/D-Met+BA (ulcerative colitis/D-methionine + butyrate). Ct group mice drank sterile water, UC group mice drank UC water (1.5% dextran sodium sulfate, 0.5% κ-carrageenan), the UC/D-Met group Imice drank 0.3% D-Met/UC water, the UC/BA (butyrate acid) group mice drank 60 ppm BA/UC water, and the UC/D-Met + BA group drank 0.3% Met and 60 ppm BA/UC water. After 2 weeks, all mice were sacrificed and blood samples, colon, liver, and spleen were collected. Colon length, liver, and spleen weight were recorded and assessed. Experimental design is shown in Figure 7.

Food and drinking water were taken freely. All mice were kept individually in a room at a temperature of 24 °C, humidity of 55%, and a 12 h light/dark cycle. This animal experiment was conducted in accordance with the “Guidelines for Animal Experiments at Nara Women’s University” and the “Standards for the Care and Storage of Laboratory Animals and the Alleviation of Pain.” (Approval No. 21-01).

### 4.2. Materials

Dextran sodium sulfate (DSS, MW 5000) was purchased from Fujifilm Wako Pure Chemicals Co. (Osaka, Japan). κ-carrageenan was purchased from Tokyo Chemical Industry Co. (Tokyo, Japan). These materials were dissolved in sterile water. D-methionine (D-Met) and butyric acid (BA) were purchased from Fujifilm Wako Pure Chemicals Corporation (Osaka, Japan). They were also dissolved in sterile water for the mice to drink. The other reagents were obtained from FUJIFILM Wako Pure Chemical Co. (Osaka, Japan).

### 4.3. Disease Activity Index

All mice were monitored for body weight and stools. The disease activity index (DAI) scored five grades of weight loss and stool consistency and four grades of occult blood by referencing Hu’s previous study [3]. The data were summed for each score of weight loss, occult blood, and stool consistency (Table 1).

### 4.4. Histopathological Analysis

Colon tissues, which were near the anus, were fixed in a 10% neutral buffered formalin solution. After that, we received paraffin embedding, thin slice, and HE (hematoxylin and eosin) staining from Genostaff, Inc (Tokyo). Histopathological preparation was observed using an OLYMPUS digital camera (CAMEDIA C-5060) with a microscope (OLYMPUS CKX41). The final magnification was ×200 and ×800. The histological scores were summed for four grades of epithelium loss, crypt damage, depletion of goblet cells, and infiltration of inflammatory cells following Kim’s previous study. Histological scores are calculated with the score of four grades as shown in Table 2.

### 4.5. Real-Time PCR Gene Expression Analysis

Total RNA was extracted from the colon, according to the RNAiso Plus laboratory manual (Takara Bio Inc., Shiga, Japan). After making a total RNA solution, the absorbance of the total RNA solution was measured and diluted with sterile water so that the concentration of extracted RNA from the colon was 0.5 µg/µL. The total RNA solutions were stored at −80 °C until required for the reverse transcription reaction.

Reverse transcription reaction solutions were made by using ReverTra Ace qPCR RT Master Mix (TOYOBO Co. LTD., Tokyo, Japan).

The reverse transcription reaction solution was mixed with the colon RNA sample (0.5 µg/µL), 5× RT Master Mix, and nuclease-free water. The reverse transcription reaction was performed according to the ReverTra Ace experimental manual. A LifePro Thermal Cycler (bulldog-bio, USA) was used for the reverse transcription reaction. The reverse transcription reaction product and complementary DNA (cDNA) solution were stored at −20 °C until the real-time PCR was performed.

RT-PCR was performed using the SYBR Green real-time PCR method on a Light Cycler Nano (Roche Diagnostics K.K., Tokyo, Japan). The PCR reaction solution was mixed with sterile water, THUNDERBIRD SYBR qPCR Mix (TOYOBO Co. LTD., Tokyo, Japan), 0.1 mM forward primer, 0.1 mM reverse primer, and cDNA solution to a total volume of 20 µL. Each primer was designed using nucleotide BLAST in NCBI and Primer synthesis was performed by FASMAC (Atsugi, Japan). The sequences of the synthesized primers are shown in Table 3.

### 4.6. Western Blot Analysis

Colon tissues were homogenized with RIPA buffer (50 mM Tris-HCl, 150 mM NaCl, 0.5% Sodium Dodecyl Sulfate, 1% NP-40, 10 mM NaF, 1 mM Phenylmethanesulfonyl fluoride) on ice and centrifuged at 15,000 g/min for 15 min (Tabletop micro-cooled centrifuge Model3500) to extract protein. After that, they were mixed with sample buffer (73.5 mM Tris-HCl, 0.2% 2-mercapto-ethanol, 10% glycerol, 3% Sodium Dodecyl Sulfate, 0.01% Bromophenol Blue). We used SDS-PAGE to separate proteins and transferred them to membranes (Immobilon-P, Merck KGaA, Darmstadt, Germany). These were blocked with 3% skimmed milk in TBST solution overnight at 4 °C. Then, incubated with primary antibodies NRF2 (nuclear factor erythroid 2-related factor 2, CUSABIO) in Solution 1 (TOYOBO, Osaka, Japan) for 1 h at room temperature. After washing the membrane with TBST, they were incubated with peroxidase-conjugated goat anti-rabbit secondary antibodies (Cell Signaling) in Solution 2 (TOYOBO, Osaka, Japan) for 1 h at room temperature. Proteins were detected by ImageQuant LAS500 (GE Healthcare Japan Com., Tokyo, Japan). Each detected band was quantified by ImageJ and the relative ratio of protein expression was analyzed using GAPDH (FUJIFILM Wako Pure Chemicals Co., Osaka, Japan) as an internal control protein.

### 4.7. Statistical Analysis

All data in this study are presented as means ± SE (standard error). Data were analyzed with GraphPad Prism version 5.0 (GraphPad Software, Inc., San Diego, CA, USA) by one-way ANOVA (analysis of variance), Dunnett vs. Ct or UC groups or two-way ANOVA Bonferroni, *t*-test, and Pearson’s correlation analysis. *p* < 0.05 was considered a statistically significant difference.

## 5. Conclusions

A mouse model of IBD has been built, which uses low molecular weight DSS and kappa carrageenan. Some evidence in this study has attempted to explain the potential mechanisms of D-methionine or butyric acid in the prevention and/or inhibition of IBD, which may identify them as good candidates for therapeutic options against IBD. In other words, our study has also demonstrated that administering D-methionine or butyric acid may have a protective effect on the development of IBD through the inhibition of oxidative stresses and/or inflammation. Mechanistically, the protective effects might be in part related to the regulation/activation of the NLRP3 and Nrf2 signaling pathway, which might be associated with anti-inflammatory effects. In conclusion, D-methionine and/or butyric acid supplementation could have a promising effect on IBD via the anti-inflammatory signaling pathway. However, it needs for further research to fully understand the molecular mechanisms involved.

## Figures and Tables

**Table 1:** Disease activity index.

Score	Weight Loss (%)	Occult Blood	Stool Consistency
0	<1	Negative	Normal
1	1–5	Occult blood stool	Soft stool
2	5–10		Loose stool
3	10–20	Bloody stool	Muddy stool
4	>20	Hematochezia	Diarrhea

**Table 2:** Histological Score.

Score	Epithelium Loss (%)	Crypt Damage (%)	Depletion of Goblet Cells	Infiltration of Inflammatory Cells
0	None	None	None	None
1	0–5	0–5	Mild	Mild
2	5–10	5–10	Moderate	Moderate
3	>10	>10	Severe	Severe

**Table 3:** Sequences of primers designed for RT-qPCR.

Gene	Forward Primer (5′→3′)	Reverse Primer (5′→3′)
*β-actin*	TTCTACAATGAGCTGCGTGTG	CTTTTCACGGTTGGCCTTAG
*IL-1β*	CCCTGCAGCTGGAGAGTGTGGA	CTGAGCGACCTGTCTTGGCCG
*TLR4*	GGCAGCAGGTGGAATTGTAT	AGGCCCCAGAGTTTTGTTCT
*MyD88*	TTGCCAGCGAGCTAATTGAGA	TTCTGTTGGACACCTGGAGA
*GSDMD*	ATCTCATTCCGGTGGACAGC	AAACACTCCGGTTCTGGTTCT
*IRAK1*	GAGACCCTTGCTGGTCAGAG	GCTACACCCACCCACAGAGT
*TRF6*	ATTTCATTGTCAACTGGGCA	TGAGTGTCCCATCTGCTTGA
*NLRP3*	ATGCTGCTTCGACATCTCCT	AACCAATGCGAGATCCTGAC

## Data Availability

Not applicable.

## Sample Availability

All compounds used in this study are commercially available.

## Abbreviations

BA	butyric acid, butyrate
D-Met	d-methionine
DSS	dextran sodium sulfate
GSDMD	gasdermin D
IBD	inflammatory bowel diseases
IL-1	βinterleukin 1β
IRAK1	IL-1 receptor-associated kinase
LPS	lipopolysaccharide
Myd88	myeloid differentiation factor 88
NLRP3	NLR family pyrin domain containing 3
Nrf2	nuclear factor-erythroid 2-related factor-2
QOL	quality of life
ROS	reactive oxygen species
SCFAs	short-chain fatty acids
TLR4	toll-like receptor 4
TRAF6	TNF receptor-associated factor 6
UC	ulcerative colitis
